# An evaluation of a liquid antimicrobial (Sal CURB®) for reducing the risk of porcine epidemic diarrhea virus infection of naïve pigs during consumption of contaminated feed

**DOI:** 10.1186/s12917-014-0220-9

**Published:** 2014-09-25

**Authors:** Scott Dee, Casey Neill, Travis Clement, Jane Christopher-Hennings, Eric Nelson

**Affiliations:** Pipestone Applied Research, Pipestone Veterinary Services, 1300 Box 188 Hwy 75S, Pipestone, MN 56164 USA; Animal Disease Research and Diagnostic Laboratory, South Dakota State University, Brookings, SD USA

**Keywords:** Porcine, Epidemic, Diarrhea, Virus, PEDV, Sal CURB®, Feed, Bioassay, Formaldehyde

## Abstract

**Background:**

Since its initial detection in May 2013, porcine epidemic diarrhea virus (PEDV) has spread rapidly throughout the US swine industry. Recently, contaminated feed was confirmed as a vehicle for PEDV infection of naïve piglets. This research provides *in vivo* data supporting the ability of a liquid antimicrobial product to reduce this risk.

**Results:**

Sal CURB® (Kemin Industries, Des Moines, IA, USA) is a FDA-approved liquid antimicrobial used to control *Salmonella* contamination in poultry and swine diets. To test its effect against PEDV, Sal CURB®-treated feed was spiked with a stock isolate of PEDV (Ct = 25.22), which PEDV-naïve piglets were allowed to ingest via natural feeding behavior (*ad libitum*) for a 14-day period. For the purpose of a positive control, a separate group of piglets was allowed to ingest non-treated (Sal CURB®-free) feed also spiked with stock PEDV (Ct = 25.22). A negative control group received PEDV-free feed. Clinical signs of PEDV infection (vomiting and diarrhea) and viral shedding in feces were observed in the positive control group 2–3 days post-consumption of non-treated feed. In contrast, no evidence of infection was observed in pigs fed Sal CURB®-treated feed or in the negative controls throughout the 14-day study period. In addition, the Sal CURB®-treated feed samples had higher (p < 0.0001) mean PEDV Ct values than samples from the positive control group.

**Conclusions:**

These data provide proof of concept that feed treated with Sal CURB® can serve as a means to reduce the risk of PEDV infection through contaminated feed. Furthermore, the results from the positive control group provide additional proof of concept regarding the ability of contaminated feed to serve as a risk factor for PEDV infection of naïve piglets.

## Background

Porcine epidemic diarrhea virus (PEDV) is an enveloped single-stranded positive sense RNA virus belonging to the Order *Nidovirales*, the family *Coronaviridae* and the genus *Alphacoronavirus* [1]. Following detection in the US swine population during May, 2013, the virus spread rapidly across the country with 6659 cases of Porcine Epidemic Diarrhea (PED) confirmed across 30 states as of May 17, 2014 [2,3]. Recently, proof of concept that contaminated feedstuffs can serve as a route of PEDV transmission to naïve pigs was reported [4]. This study evaluated the risk of PEDV-contaminated complete feed through a novel on-farm sampling method for detection of virus in feed along with an *in vivo* experiment (swine bioassay) using at-risk feed material and normal feeding behavior [4]. As this new information confirmed feed as a risk factor, it became imperative to seek solutions. Therefore, a follow up study was conducted to evaluate whether a liquid antimicrobial product containing formaldehyde and organic acid could mitigate said risk. The rationale for this approach was based on previous publications indicating that products containing formaldehyde and organic acids have a positive effect on *Salmonella* reduction in feed [5-7]. Furthermore, additional studies have demonstrated that formaldehyde treatment of organ inoculums containing Turkey Coronavirus (TCoV) rendered this material non-pathogenic, whereas other treatments failed to ameliorate its negative effects [8,9]. As PEDV and TCoV are both Coronaviruses, it was hypothesized that formaldehyde treatment of PEDV-contaminated feed may induce an anti-viral effect and prevent infection of susceptible pigs.

## Methods

### Swine bioassay facilities and source of animals

This study was conducted in Biosafety Level 2+ rooms at the Animal Resource Wing (ARW) at South Dakota State University (SDSU). All procedures involving animals throughout the study were performed under the guidance and approval of the SDSU Institutional Animal Care and Use Committee. Animals (n = 12, six-week old piglets) were sourced from a PEDV-naïve herd and were tested on arrival to the ARW via blood sampling and collection of rectal swabs from each pig. Prior to animal arrival, all rooms (walls, ceilings, floors and drains) were monitored for the presence of PEDV by PCR using sampling procedures previously described [10].

### Experimental design

Using a validated swine bioassay method [4], the 12 piglets were divided into 3 groups and each group was housed in an individual room as follows:

- **Treatment group:** Five piglets consumed feed treated with Sal CURB® and spiked with stock PEDV [11].
- **Positive control group:** Five piglets consumed saline-treated feed spiked with stock PEDV.
- **Negative control group:** Two piglets consumed feed + saline without PEDV.

### Processing of feed

Feed was sourced from a PEDV-naïve farm and screened by PCR prior to use. For the purpose of the study, a 45.5 kg allotment of feed was treated with 147.42 mL of Sal CURB®, (Kemin Industries, Des Moines, IA USA), based on an inclusion rate (per label) of 3 kg/ton of complete feed. Sal CURB® is a premix of aqueous formaldehyde solution 37% (for maintenance of complete animal feeds or feed ingredients *Salmonella*-negative for up to 21 days) and propionic acid (as a chemical preservative for control of mold in feed or feed ingredients). While Sal CURB® provides effective *Salmonella* control for up to 21 days, it is not approved for use by the U.S. Food & Drug Administration or the U.S. Department of Agriculture as a treatment for PEDV. The liquid antimicrobial was added to the feed using a syringe, injecting approximately 30 mL in 5 different locations within the 45.5 kg of feed. To promote proper mixing, the feed was stirred manually for 10 minutes using wooden spoons and strainers. Upon completion of the 10 minute mixing period, treated feed was spiked with 100 mL of a stock isolate of PEDV at a cycle threshold (Ct) value of 25.22. Twenty mL aliquots of virus were injected into 5 different locations within the feed. This level of PEDV contamination was selected based on data from actual field cases of PEDV-contaminated feed as well as levels of challenge used in the proof of concept study [4]. For the purpose of a positive control, a 45.5 kg quantity of feed was also spiked with 100 mL of stock PEDV (Ct = 25.22) along with 147.42 mL of sterile saline. Finally, feed for the negative control group was treated with 147.42 mL of sterile saline (no PEDV and no Sal CURB®). The total time required for preparation of feed batches was 60 minutes, followed by placement into the feeders of the respective rooms, allowing immediate *ad-libitum* access to feed for a 14-day period [4]. Separate mixing instruments were used to prepare the feed for all 3 groups of pigs.

### Piglet testing

Following access to treated feed, the PEDV status of all 3 groups was monitored. On a daily basis, ARW personnel inspected animals for clinical signs of PED and collected rectal swabs (Dacron swabs, Fisher Scientific, Franklin Lakes, NJ, USA) from each pig. Personnel moved from the negative control group, to the treatment group and then to the positive control group every day. Showers were taken between rooms and room-specific coveralls, footwear, hairnets, gloves and P95 masks (3 M, St. Paul, MN USA) were worn. In addition, each room was ventilated individually and HEPA filtration for both incoming and outgoing air was employed per room. If clinically affected animals were observed, swabs of diarrhea and/or vomiting, in conjunction with the daily rectal swab, were collected. Swabs were submitted to the SDSU ADRDL and tested by PCR. On day 15 of the study, animals were humanely euthanized with intravenous sodium pentobarbital and small intestinal tracts submitted for PCR and immunohistochemistry (IHC) testing and microscopic evaluation.

### Feed sampling

On 9 designated days during the study period, (days 0, 1, 3, 5, 7, 9, 11, 13 and 15), feed samples were collected from the 3 respective groups. The purpose of sampling was to document the presence of PEDV in feed and to determine whether a change in viral load occurred over time. Samples were collected from the material hopper from the feeder in each room. As these hoppers were rectangular in shape (91.44 cm deep × 61 cm long × 30.5 cm wide), protocols used to sample feed from flat-bottom trucks were referenced per the USDA Grain Inspection, Packers and Stockyards Administration [12]. For collecting feed samples, a model of a grain probe was constructed [12]. This model consisted of 2 PVC tubes (Heritage Plastics Inc., Carrolton, OH, USA), one placed inside the other per standard probe design. The outer tube was 64 cm in length with a diameter of 4.45 cm and the inner tube was 73 cm in length with a diameter of 3.18 cm. To facilitate feed entry into the lumen of the probe, seven 1.91 cm slots were drilled into each tube. Rotation of the outer tube aligned the slots across both tubes, resulting in the entry of feed into the probe via gravity flow. Once sampling was complete, the outer tube was rotated in the opposite direction, thereby closing the slots. To maintain feed in the probe lumen during sampling, as well as facilitate sample removal post-collection, both ends of the model were covered by a 3.18 cm plastic cap. Three models were constructed, one for each room.

Using the flat-bottom truck protocol, 5 samples of feed were taken from each hopper, one from each corner (n = 4) and one from the center. The goal was to collect approximately 50 grams of feed per sampling time. At each point, the probe was inserted at an angle of 0^0^ to a depth of 50 cm into the hopper, thereby “burying” the probe. During placement, the outer tube was rotated clockwise to close the slots and prevent feed entry. For sample collection, the outer tube was rotated counter-clockwise; opening the slots in both tubes and the model was moved in an up-and-down manner 2 times [12]. The outer tube was rotated to close the slots and the probe was removed. One end cap was removed and the sample was deposited into a 50 gram plastic specimen container. Once approximately 50 grams of feed were collected, a 10 mL aliquot of sterile saline was added to the specimen container. A sterile Dacron swab was inserted into sample and rotated 5 times clockwise and 5 times counter-clockwise to contact any PEDV present. The swab was removed, placed into a 3 mL plastic tube (Falcon, Franklin Lakes, NJ, USA) containing 2 mL of sterile saline and submitted for testing.

### Diagnostic procedures

All diagnostic testing was conducted using protocols developed and validated by the South Dakota State University Animal Disease Research and Diagnostic Laboratory.

### Extraction of RNA

The MagMAX™ 96 Viral Isolation Kit (Life Technologies, Waltham MA, USA) was used to obtain viral RNA from the samples, as described in the instructions provided (1836 M Revision F). A 175-μl volume of sample was used for the extraction. The magnetic bead extractions were completed on a Kingfisher96 instrument (Thermo Scientific, Waltham MA, USA).

### Real-time PCR

A commercially available real-time, single tube RT-PCR multiplex assay for the detection of PEDV and transmissible gastroenteritis virus (TGEV) was used in this study per kit instruction (Tetracore, Rockville, MD, USA). Briefly, 7 μl of the extracted RNA was added to 18 μl of the master mix. The one-step real-time RT-PCR amplification conditions started with 15 minutes at 48°C, followed by 2 minutes at 95°C. The final cycles consisted of 5 seconds at 95°C and then 40 seconds at 60°C (data collection step). The program was run for 40 cycles (Cycle time) and the FAM detector was used for PEDV and the TAMRA detector was used for TGEV. Positive and negative controls were included on each run. All amplification was completed on the ABI7500 instrumentation (Austin, TX, USA).

### PEDV stock virus propagation

For PEDV propagation, Vero 76 cells (ATCC CRL-1587) were maintained in MEM plus 10% fetal bovine serum and antibiotics. Three-day old confluent monolayers of Vero 76 cells in 150 cm^2^ flasks were washed 3 times with serum free minimum essential media (MEM) prior to inoculation. Monolayers were infected at ~0.1 moi of PEDV in MEM containing 2.5ug/ml TPCK-treated trypsin, incubated at 37°C for approximately 48 hours until obvious CPE was apparent. Flasks were frozen at −80°C until needed.

### Immunohistochemistry

Immunohistochemistry slides of porcine GI tracts were prepared using the standard SDSU ADRDL IHC procedure, with the following modification being the use of PEDV monoclonal antibody SD-6-29, of mouse ascites origin, courtesy of Steve Lawson, SDSU, at a 1:1000 dilution.

## Results

### Swine bioassay

The *in vivo* phase of the study was conducted from March 20 to April 5, 2014 (Table 1). Prior to initiation of the bioassay, all samples from incoming piglets, ARW facilities and feed were PCR negative. Throughout the 14 day study period, PEDV RNA in rectal swabs and clinical signs of PED were not observed in the treatment group or the negative control group. In addition, small intestinal tract samples from all 5 pigs in the treatment group and the 2 pigs in the negative control group were negative by PCR and IHC and no evidence of microscopic lesions of PED was observed. In contrast, in the positive control group PEDV RNA was detected in a rectal swab from the index piglet on day 2 post-ingestion (Table 1). On day 3, clinical signs of diarrhea were observed in the index piglet and another piglet was PCR positive on rectal swab. From day 4–7 post-ingestion, PEDV RNA was detected in diarrhea and rectal swabs from 3 piglets, with lethargy, vomiting and diarrhea noted. For the remainder of the 14 day study period, sporadic shedding of PEDV in feces was detected with poor condition in piglets. Small intestinal tract samples from all 5 pigs were positive by PCR and IHC. In addition, microscopic evaluation of small intestinal tissues indicated lesions indicative of PED, including re-epithelialization with diffuse villous blunting and fusion.

**Table 1:** **Summary of clinical signs and diagnostic data across the 3 groups of piglets from the swine bioassay**

	**Treatment group**		**(+) control group**		**(−) control group**	
**Days on test**	**PCR**	**Clinical signs**	**PCR**	**Clinical signs**	**PCR**	**Clinical signs**
0	Neg	Neg	Neg	Neg	Neg	Neg
1	Neg	Neg	Neg	Neg	Neg	Neg
2	Neg	Neg	19.98	Neg	Neg	Neg
3	Neg	Neg	16.32/18.84	Diarrhea	Neg	Neg
4	Neg	Neg	17.10	Diarrhea	Neg	Neg
5	Neg	Neg	16.07/15.70/16.58	V & D	Neg	Neg
6	Neg	Neg	22.13	V & D	Neg	Neg
7	Neg	Neg	21.20	Diarrhea	Neg	Neg
8	Neg	Neg	27.07	Neg	Neg	Neg
9	Neg	Neg	20.48	Neg	Neg	Neg
10	Neg	Neg	21.44	Neg	Neg	Neg
11	Neg	Neg	20.48	Neg	Neg	Neg
12	Neg	Neg	33.42	Neg	Neg	Neg
13	Neg	Neg	Neg	Neg	Neg	Neg
14	Neg	Neg	30.06	Neg	Neg	Neg
Necropsy	GI Negative	No lesions	GI positive 5/5 pigs	Lesions 5/5 pigs	GI Negative	No lesions

**Treatment group:** Pigs fed PEDV positive/liquid antimicrobial treated feed.

**(+) control group**: Pigs fed PEDV positive/saline treated feed.

**(−) control group**: Pigs fed PEDV negative/saline treated feed.

**GI Negative:** Small intestinal tracts negative by PCR & IHC.

**GI positive**: Small intestinal tracts positive by PCR & IHC.

**Lesions:** Histopathologic evidence of PEDV infection.

**DPI**: Days post-ingestion of feed.

**V & D:** vomiting & diarrhea.

### Feed testing

**Figure 1:**
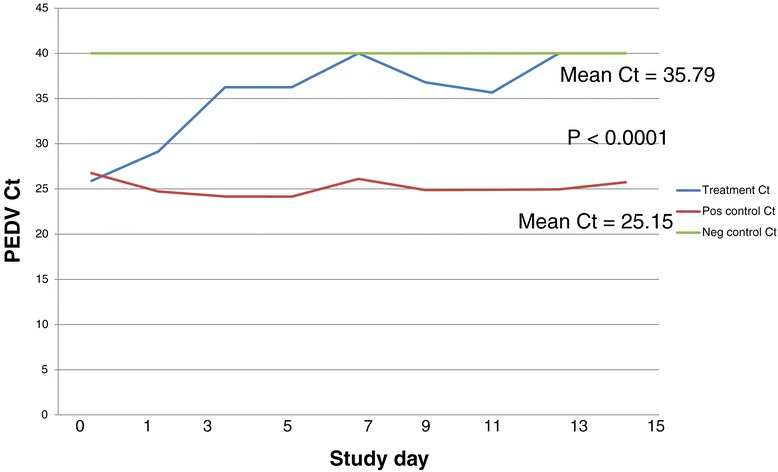
**Change in PEDV Ct levels in feed samples collected across the 3 groups over time.** This graph depicts the change over time in Ct values in the feed samples collected from each group of piglets. Note that the initial Ct levels detected in feed samples from both the treatment group and the positive control group were similar in magnitude on day 1; however, while Ct levels in feed fed to positive control piglets remained constant (red line), the levels in feed treated with the liquid antimicrobial product (blue line) demonstrate an increase over the 14 day study period. These data suggest that while viral load remained constant in non-treated feed, it decreased significantly in treated feed. Ct values from the negative control feed (green line) remained PCR negative throughout the study.

The results of the feed sampling are summarized in Figure 1 with raw data provided in Table 2. All 9 feed samples from the positive control group were positive for the presence of PEDV RNA by PCR with a mean Ct of 25.15 (range = 24.15-26.74). In contrast, the mean Ct value of the feed from the treatment group was 35.79 (range 25.89-40). When analyzed by t-test, the difference in the mean Ct levels between the treatment group and the positive control group was significant at p < 0.0001. All samples were PCR-negative from the negative control group.

**Table 2:** **Summary of PEDV Ct data from individual feed samples collected across the 3 groups over the 14 day study period**

**Sampling day**	**Treatment feed Ct**	**Positive control feed Ct**	**Negative control feed Ct**
0	25.89	26.74	40
1	29.14	24.73	40
3	38.43	24.17	40
5	36.25	24.15	40
7	40	26.11	40
9	36.79	24.87	40
11	35.67	24.91	40
13	40	24.96	40
15	40	25.74	40
**Mean Ct**	35.79*	25.15*	**40**

**Treatment group:** Pigs fed PEDV positive/liquid antimicrobial treated feed.

**(+) control group**: Pigs fed PEDV positive/saline treated feed.

**(−) control group**: Pigs fed PEDV negative/saline treated feed.

*: values significantly different at p < 0.0001.

## Discussion

Based on the results of the bioassay, Sal CURB® treated feed prevented infection and clinical disease in naïve piglets. In contrast, pigs allowed to ingest non-treated feed spiked with PEDV became infected. While both the treatment and the positive control feed contained a similar level of PEDV immediately post-processing (day 0), there was a significant difference in mean Ct at the end of the sampling period (day 15) across the 2 groups. While Ct values in treated feed changed over time, values detected in the positive control feed samples remained relatively constant. One interpretation of this observation, in conjunction with the swine bioassay data, is that the Sal CURB® product had an adverse effect of viral load and viability, while in the absence of Sal CURB® the quantity of PEDV remained constant and that virus survived over time. An acknowledged limitation was that the results are based on very small populations of pigs housed under experimental conditions and cannot be extrapolated to today’s large-scale commercial farm conditions until further testing can be conducted. In addition, the study was not designed to answer questions which still remain regarding the liquid antimicrobial product, such as the duration of activity against PEDV, its effects on other viral pathogens, its effect on dietary nutrients and the logistics of application and daily use.

In closing, this is the first publication providing evidence that a means to “biosecure” feed against a globally significant virus may be possible. Future studies should investigate whether application of the liquid antimicrobial product may have broader application at the international level and could possibly reduce the risk of the introduction of emerging and re-emerging pathogens through feed and feed ingredients that cross borders. Finally, as “feed biosecurity” is a new paradigm for the swine industry, veterinarians, producers and representatives from the feed industry will need to work together and pursue novel means to implement such a strategy.

## Conclusions

The results of this study provide initial proof of concept that the application of a liquid antimicrobial product (Sal CURB®) reduced the risk of PEDV infection through contaminated feed. Furthermore, data from the positive control group once again provide proof of concept regarding the ability of contaminated feed to serve as a risk factor for PEDV infection of naïve piglets.

## Availability of supporting data

The data set(s) supporting the results of this article is included within the article.

## Abbreviations

PEDV: Porcine epidemic diarrhea virus
PED: Porcine epidemic diarrhea
Ct: Cycle threshold
PCR: Polymerase chain reaction
ARW: Animal resource wing
FAM: Fluorescein
TAMRA: Carboxytetramethylrhodamin
MEM: Minimal essential media

## References

[1] Saif LJ, Pensaert MB, Sestak K, Yeo S, Jung K, Zimmerman JJ, Karriker LA, Ramierez A, Schwartz KJ, Stevenson GW (2012). Coronaviruses. Diseases of Swine.

[2] Chen Q, Ganwu L, Stasko J, Thomas JT, Stensland WR, Pillatzki AE, Gauger PC, Schwartz KJ, Madson D, Yoon KJ, Stevenson GW, Burrough ER, Harmon KM, Main RG, Zhang J (2014). Isolation and characterization of Porcine Epidemic Diarrhea Viruses associated with the 2013 disease outbreak among swine in the United States. J Clin Microbiol.

[3] USDA APHIS VS NVSL NAHLN UMN Swine Health Monitoring Report: *Porcine Epidemic Diarrhea Virus Reporting.* June 6, 2014.

[4] Dee S, Clement T, Schelkopf A, Nerem J, Knudsen D, Hennings J, Nelson E: **An evaluation of contaminated complete feed as a vehicle for porcine epidemic diarrhea virus infection of naïve pigs following consumption via natural feeding behavior: Proof of concept.** *BMC Vet Res* (accepted for publication). 10.1186/s12917-014-0176-9 PMC4363994 25091641

[5] Wales A, McLaren I, Rable A, Gosling RJ, Martelli F, Sayers R, Davies R (2013). Assessment of anti-Salmonella activity of commercial formulations of organic acid products. Avian Pathol.

[6] Berge AC, Wierup M (2012). Nutritional strategies to combat Salmonella in mono-gastric food animal production. Animal.

[7] Wales AD, Allen VM, Davies RH (2010). Chemical treatment of animal feeds and water for the control of Salmonella. Foodborne Pathog Dis.

[8] Brown TP, Garcia A, Kelly (1997). Spiking mortality of turkey poults: 2. Effect of six different in vitro disinfection techniques on organ homogenates capable of reproducing SMT. Avian Dis.

[9] Brown TP, Garcia A, Kelly L (1997). Spiking mortality of turkey poults: 1. Experimental reproduction in isolation facilities. Avian Dis.

[10] Lowe J, Gauger P, Harmon K, Zhang J, Connor J, Yeske P, Loula T, Levis I, Dufrense L, Main R (2014). Role of transportation in spread of porcine epidemic diarrhea virus infection, United States. Emerg Infect Dis.

[11] Nelson J, Okda F, Parmar R, Singrey A, Lawson S, Liu X, Christopher-Hennings J, Nelson E: **Environmental Stability of a Cell Culture Adapted U.S. Isolate of PEDV.** In *Proceedings of the 23*^*rd*^*International Pig Veterinary Congress.* Cancun, Mexico; 2014:94.

[12] USDA Grain inspection, Packers and Stockyards Administration: **Chapter 2: Probing and Sampling.** In *Grain Inspection Handbook.* July 7, 1995.

